# Immunity levels to poliovirus in Lao children and adults before the vaccine-derived poliovirus outbreak: A retrospective study

**DOI:** 10.1371/journal.pone.0197370

**Published:** 2018-05-15

**Authors:** Maude Pauly, Antony P. Black, Phonepaseuth Khampanisong, Phonethipsavanh Nouanthong, Judith M. Hübschen, Naphavanh Nanthavong, Kong Sayasinh, Prapan Jutavijittum, Bounthome Samountry, Anonh Xeuatvongsa, Sabine Diedrich, Claude P. Muller

**Affiliations:** 1 Infectious Diseases Research Unit, Department of Infection and Immunity, Luxembourg Institute of Health, Esch-sur-Alzette, Luxembourg; 2 Lao-Lux-Laboratory, Institute Pasteur du Laos, Vientiane, Lao People's Democratic Republic; 3 Institut de la Francophonie pour la Medecine Tropicale, Vientiane, Lao People's Democratic Republic; 4 Faculty of Medicine, Chiang Mai University, Chiang Mai, Thailand; 5 Faculty of Basic Sciences, University of Health Sciences, Vientiane, Lao People's Democratic Republic; 6 National Immunization Programme, Lao Ministry of Health, Vientiane, Lao People's Democratic Republic; 7 Department of Infectious Diseases, Robert Koch Institute, Berlin, Germany; 8 Laboratoire National de Santé, Dudelange, Luxembourg; Public Health England, UNITED KINGDOM

## Abstract

In 2015, several provinces in Lao People's Democratic Republic (Lao PDR) experienced a vaccine-derived poliovirus outbreak. This survey was conducted (i) to evaluate the vaccination coverage in different settings and cohorts using the seroprevalence of anti-poliovirus (PV) antibodies as a surrogate measure, and (ii) to explore the usefulness of an ELISA in a country with limited resources and a specific epidemiological setting. IgG antibodies were assessed by ELISA in Lao children (n = 1216) and adults (n = 1228), including blood donors and health care workers. Protective antibody titers against the 3 vaccine serotypes were determined by microneutralization (VNT) in a subset of participants. More than 92% of the children had anti-poliovirus antibodies, regardless of nutritional status or access to health care, highlighting the success of the vaccination outreach activities in the country. In contrast, anti-poliovirus seroprevalence reached only 81.7% in blood donors and 71.9% in health care workers. Participants born before the introduction of poliovirus vaccination in Lao PDR were considerably less likely to be seropositive. These findings align with the epidemiology of the outbreak. Neutralizing antibodies against at least one of the 3 poliovirus serotypes were detected in all children (99/99) and 93/99 had antibodies against all serotypes. Similarly, all health care workers had neutralizing antibodies against at least one and 92/99 against all serotypes. The comparison of both assays shows an acceptable underestimation of vaccine coverage in children by ELISA, but a low sensitivity of the ELISA in the adults. We show that the ELISA is a reasonable alternative to the VNT in particular in vaccinated children, that an improved version should be serotype specific, and that negativity thresholds should be revisited for optimal sensitivity and specificity. Thus, polio-free countries with an uncertain vaccination coverage and limited laboratory capacity, that are at risk of vaccine-derived poliovirus outbreaks or of re-importation of wild poliovirus may benefit from an improved ELISA for cohort studies to evaluate their immunization program in children.

## Introduction

Large-scale vaccination campaigns eliminated wild poliovirus (WPV) in most countries of the world and WPV remains endemic only in Pakistan, Nigeria and Afghanistan [1]. In the Lao People’s Democratic Republic (Lao PDR), oral polio vaccine (OPV) was introduced in the early eighties, and vaccination was expanded to the whole country in the nineties [2]. In 1996, the last case of WPV was reported in Lao PDR and in 2000, the Western Pacific Region, including Lao PDR, was certified polio-free [3, 4].

Despite its ground-breaking role in the eradication of poliovirus (PV), OPV (containing live attenuated PV strains) has some important drawbacks. A small proportion of vaccinees develop vaccine-associated paralytic poliomyelitis (VAPP, reviewed in [5]). In countries with suboptimal vaccination coverage levels and weak acute flaccid paralysis (AFP) surveillance, excreted vaccine-virus can replicate and circulate for a prolonged time. Within less than a year of circulation, vaccine viruses may accumulate genetic mutations and neurovirulent vaccine-derived PV (VDPV) may emerge [6].

**Fig 1:**
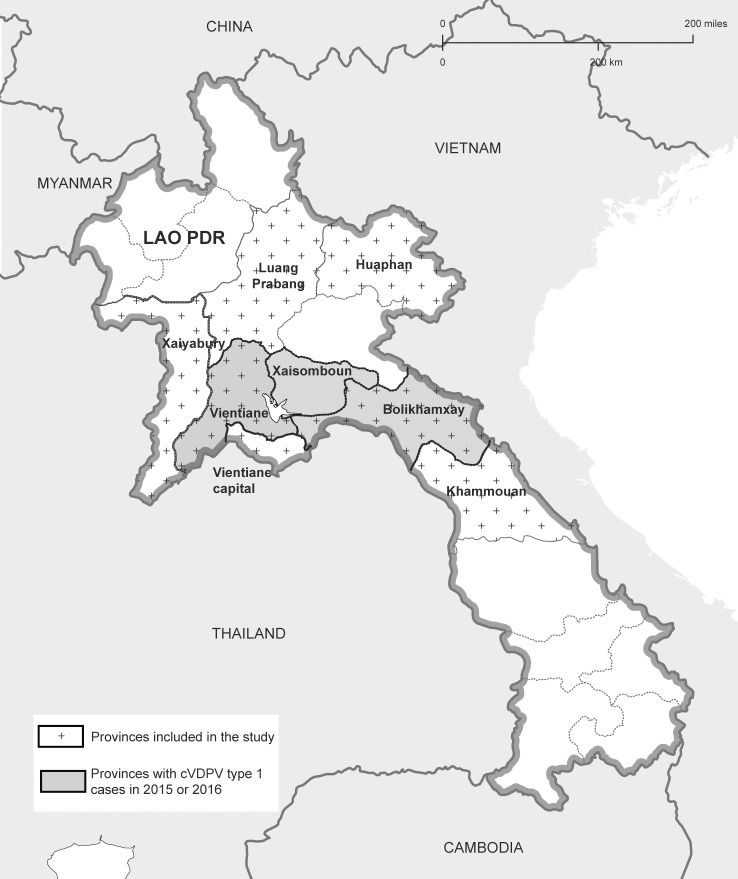
Lao provinces. Provinces affected by the cVDPV type 1 outbreak are represented in grey and provinces included in the study are marked with crosses. Reprinted from *Political map of Laos* under a CC BY license, with permission from [www.onestopmap.com], original copyright 2015.

At the end of 2015, VDPV type 1 strains emerged in Lao PDR and caused paralysis in 11 individuals (last case in January 2016) in the three neighbouring provinces Bolikhamxay, Xaisomboun and Vientiane Province (see also Fig 1). Most of the affected patients were male; 4 were below the age of 18 months, 4 between 4 and 15 years, and 2 were in their forties. Only a one-year old child had completed the three OPV vaccinations. The others had only one or no dose of OPV. Virtually all belonged to the ethnic group of the Hmong. Circulation of the VDPV (cVDPV) was confirmed by epidemiological investigations and molecular analyses. By the beginning of 2016, the outbreak was declared a public health emergency by the Prime Minister and large vaccination campaigns with trivalent OPV were launched and surveillance was intensified throughout the country [7, 8].

To reduce the risk of cVDPV emergence and in compliance with the Polio End Game Strategy [9], a switch from trivalent (containing type 1, 2 and 3 vaccine strains) to bivalent (containing type 1 and 3 vaccine strains) OPV was globally implemented in 2016 [9–11]. In Lao PDR, the new recommendations, introduction of bivalent OPV and of Inactivated polio vaccine (IPV) as an adjunct to routine immunization with OPV will soon be implemented.

Currently, PV surveillance relies mainly on the reporting of AFP and in Lao PDR, reported rates of AFP reached almost the recommended minimum required for PV-free countries (i.e.one per 100 000 children below 15 years of age) [12]. However, PV surveillance remains challenging as silent circulation of WPV and VDPV is frequent. The microneutralization assay (VNT) is well recognized as the gold standard for measuring neutralizing antibodies against PV and as a correlate of protection [13]. Therefore, it is also the best assay for assessing population immunity. However, the assay is time- and resource-consuming, and major restrictions apply to its use. Thus the capacity to perform population or cohort studies with this assay is limited even in fully accredited polio laboratories. Only recently, storage and handling of type 2 PV has been prohibited even in most polio reference laboratories. In the future, working with live PV will be subject to further restrictions in particular when poliomyelitis will be eradicated. Also, not all sera collected under difficult field conditions may be suitable for VNT (e.g. sterility, quantities). Moreover, countries and courier services become increasingly reluctant to ship specimen to specialized laboratories and costs are often prohibitive. With the global introduction of injectable IPV into national immunization programs, the relative importance of serum IgG detection will likely increase. In contrast to the VNT, an ELISA can be performed with little limitations in most laboratories. Therefore it could be a simple and valuable alternative to the very limiting VNT, complementing the VNT in the polio endgame.

Not enough is known in Lao PDR about immunity levels against PV to understand the reasons for the recent VDPV outbreak. We investigated five large cohorts with different demographic, exposure and vaccination histories. As Lao PDR has no laboratory to perform VNT, this assay was done at the Robert Koch Institute in Berlin. Our study explored the usefulness of a commercial polio IgG ELISA as a surrogate measure of vaccination coverage and provides important public health information about polio epidemiology.

## Material and methods

### Participants

Serum samples from 5 different cohorts, collected before the 2015 cVDPV outbreak for seroprevalence studies on infectious diseases, were included. We selected our cohorts from urban or rural areas of the provinces affected by the recent VDPV type 1 outbreak (Bolikhamxay, Vientiane Capital and Xaisomboun) or provinces neighboring the latter. Both, children and adults, with known and unknown vaccination status, as well as health care workers (HCW) as a risk group in the case of an outbreak were included. Although all cases occurred in an ethnic minority from remote areas, ethnicity was for ethical reasons not captured in our study. For each participant, birth year and sex were available. Serum and data collection were performed anonymously after obtaining written informed consent by the participant or the participant's legal guardian. The studies were approved by the National Ethics Committee for Health Research of the Ministry of Health in Lao PDR (Ethical approval reference NECHR 2013–860, 2013–732, 2014–059, 2013–038 and 2017–016).

#### Fully vaccinated children (Cohort 1)

This cohort included 806 children, aged less than 3.5 years. All children had complete Health Center records of three doses of pentavalent vaccine and of OPV. Until May 2016, the 3 doses of OPV were given together with the pentavalent vaccine at weeks 6, 10 and 14. The presence of antibodies against tetanus as determined in a previous study [14] was used as a proxy for attending the vaccination session. In 2013 and 2014, samples were collected in 2 provinces with VDPV type 1 cases in 2015/2016 (Bolikhamxay and Vientiane) and in an adjacent province (Khammouane) (Fig 1). The following nutritional indicators were calculated: WHZ (weight-for-height Z-scores), HAZ (height-for-age Z-scores) and WAZ (weight-for-age Z-scores). Moreover, birthplace (i.e. home or hospital) was recorded (Table 1).

**Table 1:** Association between socioeconomic characteristics and anti-polio IgG seropositivity as determined by ELISA for each study cohort.

# Cohort	Predictor (significance level)	Categories	% of Total	Anti-Polio seroprevalence	
				n (%)^a^	n (%)^b^
#1 Fully vaccinated children (N = 806)	Total Cohort		100	787/806 (97.6)	757/776 (97.6)
	Province (**)	Bolikhamxay	26.1	199/210 (94.8)	188/199 (94.5)
		Khammouane	42.9	345/346 (99.7)	338/339 (99.7)
		Vientiane	31	243/250 (97.2)	231/238 (97.1)
	Age group	≤ 1 year	8.9	70/72 (97.2)	69/71 (97.2)
		1 year > x ≥ 2 years	52.1	408/420 (97.1)	391/403 (97)
		2 years > x ≥ 3.5 years	39	309/314 (98.4)	297/302 (98.3)
	Sex	Female	51.4	405/414 (97.8)	389/398 (97.7)
		Male	48.6	382/392 (97.5)	368/378 (97.4)
	Birthplace	Home	30	239/242 (98.8)	230/233 (98.7)
		Hospital	70	548/564 (97.2)	527/543 (97.1)
	WHZ	≥ -2	89.1	699/718 (97.4)	672/691 (97.3)
		< -2	8.1	65/65 (100)	62/62 (100)
		Unknown	2.8	N/A	N/A
	HAZ	≥ -2	57	448/459 (97.6)	430/441 (97.5)
		< -2	39	307/315 (97.5)	297/305 (97.4)
		Unknown	4	N/A	N/A
	WAZ	≥ -2	78.5	616/633 (97.3)	592/609 (97.2)
		< -2	20.8	166/168 (98.8)	160/162 (98.8)
		Unknown	0.7	N/A	N/A
#2 Children from remote areas (N = 90)	Total Cohort		100	88/90 (97.8)	84/85 (98.8)
	District	Xam Tai	58.9	51/53 (96.2)	49/50 (98)
		Kuan	41.1	37/37 (100)	35/35 (100)
	Age group	<2 years	16.7	15/15 (100)	15/15 (100)
		2 years ≥ x > 3years	22.2	19/20 (95)	18/19 (94.7)
		3 years ≥ x ≥ 5 years	61.1	54/55 (98.2)	51/51 (100)
	Sex	Female	43.3	39/39 (100)	36/36 (100)
		Male	56.7	49/51 (96.1)	48/49 (98)
#3 Children with unknown vaccination status (N = 320)	Total Cohort		100	297/320 (92.8)	277/300 (92.3)
	Province (*)	Bolikhamxay	30.6	84/98 (85.7)	74/88 (84.1)
		Luang Prabang	43.8	134/140 (95.7)	124/130 (95.4)
		Vientiane	25.6	79/82 (96.3)	79/82 (96.3)
	Age group (**)	≤ 1 year	39.1	122/125 (97.6)	122/125 (97.6)
		5 years ≥ x ≥ 9 years	60.9	175/195 (89.7)	155/175 (88.6)
	Sex	Female	51.3	152/164 (92.7)	146/158 (92.4)
		Male	48.7	145/156 (93)	131/142 (92.3)
#4 Blood donors (N = 528)	Total Cohort		100	441/528 (83.5)	389/476 (81.7)
	Province	Huaphan	15.3	67/81 (82.7)	60/74 (81.1)
		Khammouane	48.7	215/257 (83.7)	185/227 (81.5)
		Vientiane	25.8	113/136 (83.1)	104/127 (81.9)
		Xaiyabury	10.2	46/54 (85.2)	40/48 (83.3)
	Birthyear group (**)	1989–1998	50.4	230/266 (86.5)	206/242 (85.1)
		1978–1988	32.4	144/171 (84.2)	129/156 (82.7)
		1958–1977	17.2	67/91 (73.6)	54/78 (69.2)
	Sex	Female	38.8	175/205 (85.4)	162/192 (84.4)
		Male	61.2	266/323 (82.4)	227/284 (80)
#5 Healthcare workers (N = 700)	Total Cohort		100	536/700 (76.6)	420/584 (71.9)
	Location hospital	Central or provincial	72.7	388/509 (76.2)	304/425 (71.5)
		District	27.3	148/191 (77.5)	116/159 (73)
	Birthyear group	1989–1998	12.4	72/87 (82.8)	59/74 (79.7)
		1978–1988	29.3	160/205 (78.1)	130/175 (74.3)
		1958–1977	58.3	304/408 (74.5)	231/335 (69)
	Sex	Female	78.7	429/551 (77.9)	335/457 (73.3)
		Male	21.3	107/149 (71.8)	85/127 (66.9)
	Position	Lab technician	7.3	41/51 (80.4)	35/45 (77.8)
		General physician	21	112/147 (76.2)	90/125 (72)
		Nurse	53.9	287/377 (76.1)	219/309 (70.9)
		Others	12.1	64/85 (75.3)	51/72 (70.8)
		Specialist physician	5.7	32/40 (80)	25/33 (75.8)

^a^Complete dataset, borderlines being considered positive

^b^Dataset without borderline samples

*significant effect on anti-poliovirus antibody seroprevalence (p-value between 0.05 and 0.01)

**highly significant effect on anti-poliovirus antibody seroprevalence (p<0.01)

#### Children from remote areas (Cohort 2)

In 2013, a total of 90 children aged less than 5 years were recruited in two districts (Xam Tai and Kuan) from Huaphan province. This poor and hard-to-reach province borders on Xaysoumboun which reported VDPV type 1 cases in the recent outbreak (Fig 1). Distance to the next health care facility (greater or less than 100 minutes by the most rapid means of transportation) was registered as proxy for access to health care (Table 1).

#### Children with unknown vaccination status (Cohort 3)

In 2012, 320 children aged less than 9 years and with unknown OPV status were recruited from 3 provinces (i.e. Bolikhamxay, Vientiane and Luang Prabang) (Fig 1).

#### Blood donors (Cohort 4)

This cohort from 2014 included 528 blood donors, aged 16 to 56 years, and with unknown vaccination status from 4 provinces (i.e. Vientiane, Huaphan, Khammouane and Xaiyabury) (Fig 1).

#### Healthcare workers (Cohort 5)

This cohort included 700 HCW aged between 15 and 69 years and with different clinical and non-clinical positions. Sample collection took place in 2013, in 3 central, 2 provincial and 8 district hospitals located in Vientiane capital, Huaphan and Bolikhamxay provinces (Fig 1).

### Laboratory analysis

IgG antibodies against the three PV types were detected using a Poliovirus IgG ELISA kit (Immuno-Biological Laboratories-America, Minnesota, USA) according to the manufacturer’s instructions. Samples with antibody levels of 10U/mL±20% were considered borderline. This ELISA assay detects neutralizing and non-neutralizing antibodies and is not suitable for intra-typic (i.e. vaccine-derived or wild-type virus) and inter-typic (i.e. PV types 1–3) differentiation of poliovirus antibodies. Neutralizing antibodies against the 3 vaccine strains (Sabin 1–3) were determined by VNT at the Polio Reference Laboratory at the Robert Koch Institute in Berlin, following the WHO protocol [13]. Only partial antibody titration was performed and the antibody titer cutoff for protection was 8. The VNT was performed only on a subset of the samples (n = 198; 99 of Cohort 1 and 99 of Cohort 5) since the Robert Koch Institute did not have the capacity to test all cohorts and specimens for study purposes. While restrictions applied for performing VNT in our fully equipped BSL2/BSL3 laboratory, there were no such limitations for performing ELISAs.

### Statistical analyses

Statistical analyses were performed in R software (version 3.3.1.; R Foundation for Statistical Computing, Vienna, Austria) [http://www.R-project.org] using the packages ‘MASS’ [15], ‘fmsb’[http://CRAN.R-project.org/package=fmsb], ‘epitools’ [http://CRAN.R-project.org/package=epitools], ‘aod’ [http://cran.r-project.org/package=aod], ‘ggplot2’[16], ‘car’ [17], ‘cowplot’ [https://CRAN.R-project.org/package=cowplot] and ‘Rcpp’ [18]. We observed little difference in results whether borderline samples (10U/ml±20%) were considered positive or were ignored (Table 1). Our statistical analysis was based on a dataset without borderline samples. Univariate, bivariate and multivariate analyses were conducted to assess factors associated with immunization status (i.e. seropositive or seronegative). We used two-tailed Fisher’s exact test or Yates’s corrected χ^2^ test, as appropriate, to compare categorical variables and t-test to compare quantitative variables. Binary logistic regression was applied to estimate the probability of PV seropositivity given the values of following explanatory variables: age, birth year, sex, birthplace, or province. Pearson correlation coefficient was calculated to measure the association between anti-PV antibody levels and age, and between anti-PV antibody levels determined by ELISA and neutralizing antibody titers determined by VNT. A p-value less than 0.05 was considered statistically significant and a p-value less than 0.01 was considered highly significant.

## Results

### Seroprevalence in children (Cohorts 1–3)

97.6% of the children with full vaccination records and aged less than 3.5 years had IgG antibodies against PV by ELISA (Cohort 1). Neither sex or age, nor birthplace or nutritional status influenced PV immune status. However, seropositivity rates differed significantly between the 3 provinces (χ^2^ = 14.7, df = 2, p<0.01; Table 1).

An anti-PV antibody seroprevalence of 98.8% was determined by ELISA in children aged less than 5 years from remote districts in Huaphan (Cohort 2). Neither age or sex, nor distance to next health care facility had a significant effect on PV immune status (Table 1). In another cohort of children with unknown vaccination status from less marginalized communities (Cohort 3), antibody seroprevalence was somewhat lower with 92.3%. Similarly to what was found for cohort 1, significantly lower immunity rates were also found for this cohort in Bolikhamxay (84.1%) than in the two other provinces (Luang Prabang: 95.4%, OR = 3.9, 95%CI = 1.4–10.6, p = 0.01; Vientiane: 96.3%, OR = 4.9, 95%CI = 1.4–18, p = 0.007). Children aged less than 1 year were significantly more likely to have anti-PV antibodies than children aged 5 to 9 years (97.6% versus 88.6%, p = 0.007) and anti-PV antibody levels were negatively correlated with age (Pearson's r = -0.4; 95%CI = -0.5 and -0.3; t = -7.1, df = 318, p<0.001).

### Seroprevalence in adults (Cohorts 4 and 5)

**Fig 2:**
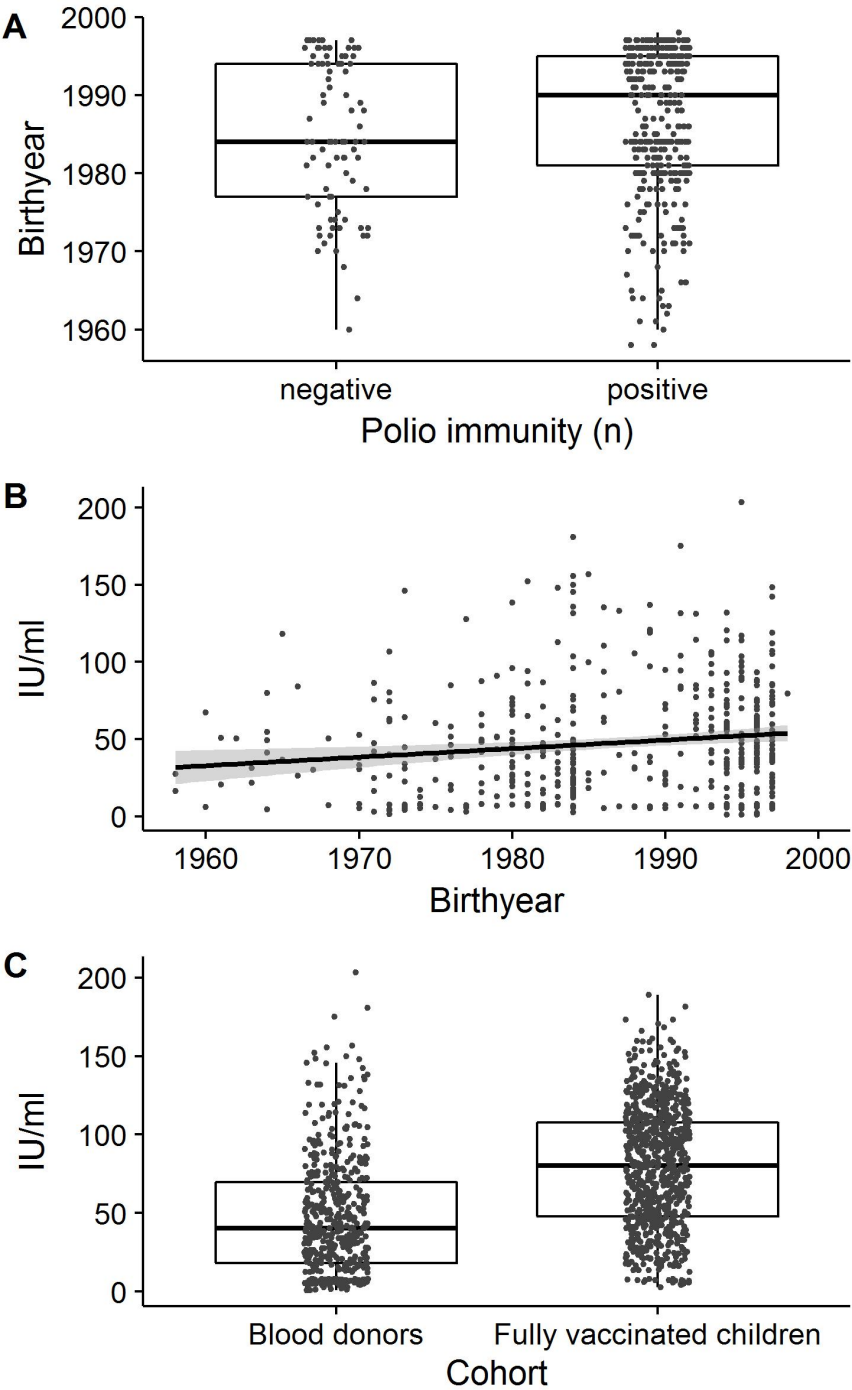
Age-related differences in poliovirus immunity as determined by ELISA. (A) Differences in age between seronegative and seropositive blood donors and (B) age-stratified anti- poliovirus antibody levels in blood donors. The regression line and the confidence interval (shaded) are shown. (C) Differences in median antibody levels between fully vaccinated children (Cohort 1) and blood donors (Cohort 4).

81.7% of the blood donors (Cohort 4) had antibodies against PV by ELISA. Seroprevalence rates ranged from 81.1 to 83.3% in the 4 provinces with no significant differences. Participants born after the introduction of OPV into the national immunization program in 1979 were significantly more likely to be seropositive: only 69.2% of the birth cohort 1958–1977 were seropositive, compared to 82.7 and 85.1% of the younger birth cohorts (1978–1988: OR = 2.1, 95%CI = 1.1–4, p = 0.028; 1989–1998: OR = 2.5, 95%CI = 1.4–4.6, p = 0.003) (Table 1, Fig 2A). Anti-PV antibody levels correlated negatively with age (Pearson's r = -0.1; 95%CI = -0.2 and 0; t = -3.6, df = 526, p = 0.001) (Table 1, Fig 2B). Mean antibody levels were significantly lower in blood donors (47.6, 95%CI = 18.2–69.6) than in the fully vaccinated children (79.1; 95%CI = 47.7–79.1) (t = -14.3, df = 1031.5, p-value<0.001; Fig 2C).

Overall, a lower seroprevalence was determined for HCW (Cohort 5) than for blood donors: only 71.9% of the HCW had antibodies against PV by ELISA. Also in this adult cohort, the antibody seroprevalence decreased with age from 79.7% (birth year 1989–1998), to 74.3% (birth year 1978–1988), to 69% (birth year 1958–1977), but between the youngest and the oldest cohort there was only a trend to significance (p = 0.067). Anti-PV antibody levels were negatively correlated with age (Pearson's r = -0.2; 95%CI = -0.2 and -0.1; t = -2.9, df = 698, p-value = 0.004). The other recorded risk factors wee rnot significant (Table 1).

### Performance characteristics of the ELISA compared to the VNT assay

Subsets of Cohort 1 (n = 99) and Cohort 5 (n = 99) were tested by both, VNT and ELISA. Regression analysis showed no significant correlation between the antibody titers determined by VNT and the antibody levels (in arbitrary units) determined by ELISA. While 80.8% of the fully vaccinated children of this Cohort 1 subset were positive for anti-polio IgG antibodies by ELISA, essentially all had protective antibodies against PV types 1 and 2 (99%) and against PV type 3 (94.9%) by VNT. 93.9% of the children had protective antibodies even against all three PV types (Table 2). In the HCW subset (Cohort 5), 49.5% were positive for anti-polio IgG antibodies by ELISA, but essentially all had protective antibodies to PV type 2 (99%) and PV types 1 and 3 (97%) by VNT. 92.9% of the HCW had protective antibodies to all three PV types (Table 2).

**Table 2:** Sensitivity of the ELISA against the microneutralization assay.

		*Neutralizing antibodies against the 3 PV types (by VNT)*			
		PV type 1	PV type 2	PV type 3	PV type 1–3
		n (%)	n (%)	n (%)	n (%)
**#1 Fully vaccinated children (N = 99)**	*Total cohort*	98 (99.0)	98 (99.0)	94 (94.9)	93 (93.9)
	*Anti-PV IgG antibodies (80*.*8% seropositivity by ELISA)*	80 (81.6)	79 (80.6)	78 (83.0)	77 (82.8)
**#5 Healthcare workers (N = 99)**	*Total cohort*	96 (97.0)	98 (99.0)	96 (97.0)	92 (92.9)
	*Anti-PV IgG antibodies (49*.*5% seropositivity by ELISA)*	48 (49.0)	49 (50.0)	47 (50.0)	46 (49.5)

**Fig 3:**
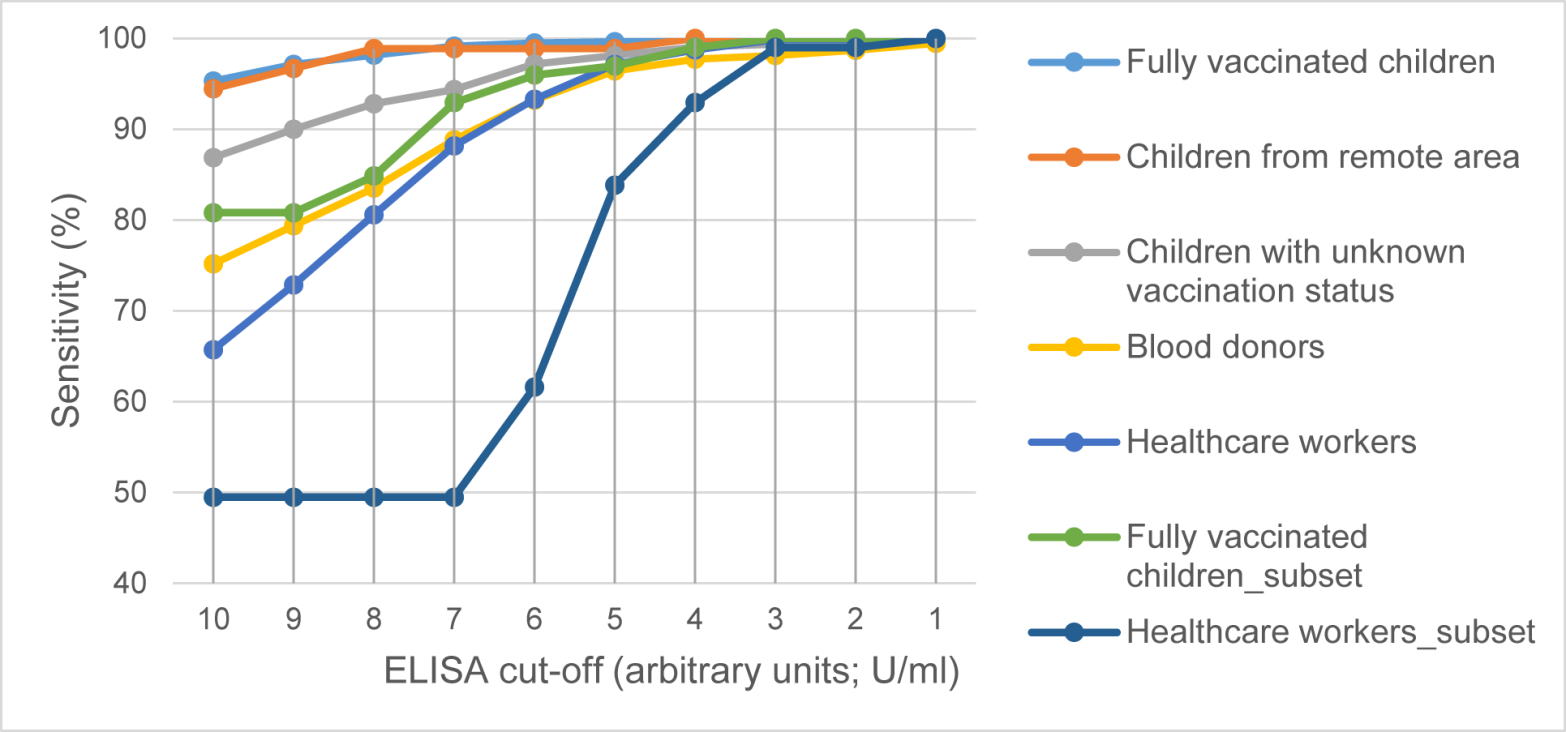
Technical sensitivity of the ELISA relative to a moving cut-off for seropositivity. The dashed lines represent the sensitivity of the subset of samples tested by VNT of both Cohorts 1 and 5 (Fully vaccinated children and Healthcare workers). The solid line corresponds to the sensitivity of the complete Cohorts 1–5.

Since every child and HCW had neutralizing antibodies against at least one PV type, the ELISA underestimated the “vaccination coverage” lacking some sensitivity: At the cut-off proposed by the manufacturer (10U/mL±20%), the sensitivity for Cohort 1 was technically 80.8% while the sensitivity to predict antibodies against any particular PV type or against all 3 PV types was 80.6 to 83% (Table 2). The corresponding sensitivities for Cohort 5 ranged between 48.9 to 50% (Table 2). If the cut-off was lowered to the lower limit of the grey-zone (8 U/ml) the sensitivities increase from 97.6% and 76.6% to about 98.1% and 80.6% for both cohorts respectively (Fig 3). At a cut-off of 7U/ml, i.e. slightly below the grey-zone the sensitivity of the ELISA would reach >99% in the child cohort 1 and an acceptable 88.1% in the adult Cohort 5. Assuming that also all participants of Cohort 2, 3, and 4 are positive for at least one PV serotype, the sensitivities for these cohorts would increase from 97.8%, 92.8% and 83.5% to 98.9%, 94.4%, and 88.8%, respectively. However, the specificity and the effect of lowering the positivity threshold on specificity could not be evaluated since there were no VNT-negatives for all 3 PV types available.

## Discussion

To reduce the risk of WPV re-importation in Lao PDR [19, 20], the immunization services were strengthened nationwide [21]. In 2015, the OPV vaccination coverage was estimated at 89% for 12–23 months old children [22]. Nevertheless, in the same year, the country experienced a cVDPV type 1 outbreak.

We find here that overall well above 90% of children had antibodies against PV. This was true even for a rural cohort of children with unknown vaccination status (Cohort 2). This high seroprevalence is also largely due to a very high vaccine efficacy: 97.6% of fully vaccinated children (Cohort 1) were positive for anti-PV antibodies by ELISA. Although weak vaccine responses to OPV have been reported in chronically malnourished children [23], we observed no effect of malnutrition on antibody levels. Also no significant difference in PV immunity was found between children born at home and children born in hospital settings. Remarkably, the cohort of children from remote districts in Huaphan were equally well protected as the fully vaccinated children, and distance to the next health care facility had no negative impact on their immune status. This suggests a high efficiency of outreach vaccination activities. However, significantly lower seropositivity rates were determined in Bolikhamxay, both for fully vaccinated children (94.5%) and children with unknown OPV status (84.1%). This is also the province which notified the first paralytic cases during the outbreak. Deficiencies related to vaccine management may also have influenced OPV efficacy [24–26].

In our adult cohorts, we found that only 81.7% of the Lao blood donors and 71.9% of the HCW had anti-PV antibodies by ELISA. There was no significant difference between blood donors from different provinces and between HCW from central, provincial or district hospitals or with different clinical and non-clinical positions. In both adult cohorts, older participants and particularly those born before the introduction of OPV in the country had significantly lower anti-PV antibody levels and, were significantly less likely to be seropositive (Fig 2A and 2B) than younger adults or the children of Cohort 1 and 2. Adults born before the eighties did not undergo routine vaccination and were also less likely to participate in SIAs. The significant age-dependent decrease in total anti-PV antibodies determined by ELISA among all adult cohorts could thus be a reflection of the lower vaccination coverage in this age group, or of the waning immunity after vaccination or exposure to VDPV or WPV. This may also explain why four of the eleven reported paralytic cases occurred among individuals above the age of 14 years.

As mentioned before, the gold-standard for testing immunity is the VNT. Because of its restricted use, we could test only selected subsets of samples from two cohorts: The cohort of children (Cohort 1) with documented recent vaccination was assumed to be most likely to have antibodies against PV, allowing to test the sensitivity of the assays against vaccination records. Conversely, this cohort also served to measure the seroconversion i.e. efficacy of recent vaccination activities. The cohort of health care workers (Cohort 5) was selected as an example of an adult population. In addition, it represents a group with a particular risk in the case of an outbreak. Whereas Cohort 1 was largely ELISA positive, the latter cohort had a sizable proportion of ELISA negatives, allowing to test also negatives by VNT. The ELISA serologies of the other children’s cohorts (Cohort 2, 3) and the other adult cohort (Cohort 4) matched the results of Cohort 1 and cohort 5, respectively. Thus, Cohorts 1 and 5 were adequate choices for being tested by VNT.

The VNT results were similar in both subsets with all cohort members having antibodies at least against one PV type, demonstrating that both the vaccination coverage and the vaccine response are highly satisfactory. Nevertheless, only 49.5% of the adult subset and 80.8% of the child subset were positive by ELISA. This shows a significant lack of sensitivity of the ELISA compared to the VNT in both cohorts. The sensitivity of the ELISA could be improved by lowering the threshold for positivity below the recommended threshold, albeit normally at the expense of more false positives, i.e. a reduced specificity. Because all specimens tested by VNT had antibodies against at least one PV type, it was not possible to estimate the specificity of this trivalent ELISA against the monovalent VNTs in the Lao setting. Interestingly, the vast majority of vaccinated children (93.9%) and of the adults with unknown vaccination status (92.9%) tested by VNT had protective antibodies against the 3 PV types. Thus, overall, 87.1–91.8% of the children and 71.2–77.6% of the adult cohort would have antibodies against the three PV types.

The discrepancy between the two assays, particularly conspicuous in the adults born before (37.9%) and after (58%) the introduction of OPV in the early eighties may be explained by the lower vaccination coverage rates or waning of antibodies after WPV or VDPV exposure. While neutralization is a reliable correlate of protection [24], Lao adults born before OPV introduction may be sufficiently protected against disease, but not necessarily against infection [25]. Silent spreading of PV [26, 27] may favor the re-emergence of cVDPV in particular in high-risk areas. While an overestimation would not be tolerable, the ELISA used provided an acceptable underestimation of the vaccine coverage in children. Thus, countries with an uncertain vaccination coverage and/or vaccine response, that are at risk of VDPV or re-importation of WPV and/or plan to switch from OPV to IPV and that cannot perform VNT, may benefit from an improved version of such an ELISA for cohort studies to evaluate their immunization programme in children. Our results show that in settings that were declared polio-free since time of vaccination of the cohort, antibodies can largely be attributed to the trivalent vaccination, in particular if they are directed against the three PV types, as we have shown by VNT. As Lao PDR is polio-free since 2000, seropositivity by ELISA is highly indicative of vaccination with trivalent OPV and not of exposure to cVDPV or WPV. Nevertheless an improved ELISA should be serotype specific and negativity thresholds should be revisited for optimal sensitivity and specificity. The value of measuring different isotypes and isotype subclasses including secretory IgA should be explored [28, 29].

## Conclusions

Thus, our seroprevalence results are in line with what was observed during the outbreak. However, a limitation of our cohorts was that for ethical reasons we were unable to collect explicit information on ethnicity, while ethnic minority communities were most affected by the outbreak. These and adult risk groups such as HCWs, and regions with weak vaccination programs should be primarily targeted by future supplementary vaccination activities.
